# Individual‐Level Drivers of Food Choices and Diet Quality Among Adolescents in Urban West Africa: Evidence From Accra, Ghana

**DOI:** 10.1111/mcn.13775

**Published:** 2024-12-09

**Authors:** Janosch Klemm, Samuel Muli, Kolade Oluwagbemigun, Martin Parlasca, Aba Crentsil, Deda Ogum, Peter Quartey, Amos Laar, Anna Lartey, Christian Borgemeister, Ute Nöthlings

**Affiliations:** ^1^ Center for Development Research (ZEF) University of Bonn Bonn North Rhine‐Westphalia Germany; ^2^ Institute of Nutrition and Food Sciences (IEL) ‐ Nutritional Epidemiology University of Bonn Bonn North Rhine‐Westphalia Germany; ^3^ Institute of Statistical, Social and Economic Research University of Ghana Accra Greater Accra Ghana; ^4^ Department of Population, Family & Reproductive Health, School of Public Health University of Ghana Accra Greater Accra Ghana; ^5^ Department of Nutrition and Food Science University of Ghana Accra Greater Accra Ghana

**Keywords:** adolescents, diet diversity, diet quality, Ghana, Global Dietary Recommendation (GDR) Score, urban settings

## Abstract

Diet quality is influenced by multiple individual factors, but their relative strength and importance remain unclear. We investigate the associations between five domains of individual factors (economic, cognitive, aspirational, situational and consumer behaviour) and diet intake of adolescents in Accra, Ghana. A cross‐sectional survey among Junior High School (JHS) students (*n* = 409, mean age 14.3 years ± 1.28 (SD)) in Accra, Ghana, was conducted. Data on diet intake, knowledge, attitude and practices (KAP) and socioeconomic background characteristics were collected. Adjusting for other factors, students' total budget was positively associated with food group diversity (*β* = 0.12, 95% confidence interval [CI] 0.09–0.15) but inversely associated with diet quality (*β* = −0.07, 95% CI −0.11 to −0.03). Positive attitude towards nutrition and healthy eating was inversely associated with unfavourable diversity (*β* = −0.17, 95% CI −0.31 to −0.03). Differences between negative deviants relative to positive deviants were determined by attitude towards healthy eating (odds ratio [OR] = 0.41; 95% CI 0.17–0.99) and family practices (OR = 0.48; 95% CI 0.23–1.00). We provide evidence that higher food budgets were associated with higher diet diversity, but not with improved diet quality. Attitude, but not knowledge, was linked to better diet quality. Future studies should focus on the specific contribution of aspirational, situational and behavioural factors in directing increased diversity towards favourable eating habits.

## Summary

- Adolescents with higher budgets tended to consume more unfavourable food groups, relative to favourable food groups.
- No budget difference was found between positive deviants (Nutrition Champions) versus negative deviants (Nutrition Challengers). They differ mainly regarding attitudes and family practices.
- Attitudes, such as perceived benefits of healthy eating, were identified as the most relevant mitigating factor for the intake of *unfavourable* food groups. Practices, especially family practices such as regularly eating home‐cooked meals, on the other hand, were associated with the intake of *favourable* food groups.
- These specific pathways of individual‐level factors must be considered when informing policies or programmes for adolescent diet intake.

## Graphical abstract

We investigate how different individual factors are associated with diet quality in adolescents. A higher budget is associated with higher food group diversity, but not better diet quality. Eating more *favourable* food groups—and improving diet quality—is associated with budget, improved attitudes and better (family) practices.

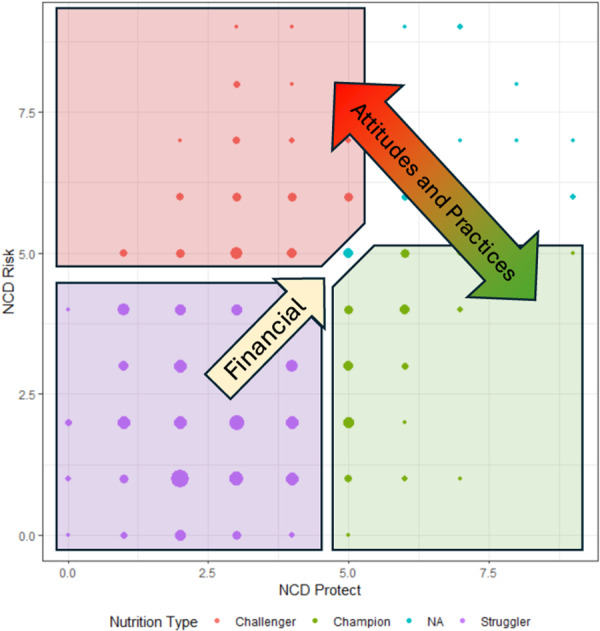

## 1 Introduction

People's dietary choices have substantial impacts on individual health, public health and planetary health (Afshin et al. 2019; Qiao et al. 2022; Springmann et al. 2018). However, the determinants of these choices are not well understood, particularly for vulnerable demographic groups such as adolescents in low‐ and middle‐income countries (LMICs) (Fanzo and Davis 2021; Kupka, Siekmans, and Beal 2020). Adolescence, a critical period in life, is marked by susceptibility to various forms of malnutrition (Norris et al. 2022), establishments of dietary patterns that often persist into adulthood, influencing long‐term health outcomes and predisposing individuals to chronic disease risk (ALjaraedah, Takruri, and Tayyem 2019; Contento et al. 2006; Cutler et al. 2011; Tabbakh and Freeland‐Graves 2016).

As a typical sub‐Saharan African metropolitan area, Accra, the capital of Ghana, is characterised by increasing urban sprawl, rising income and employment levels, increased demand for convenient shopping and high availability of ultra‐processed foods (Mockshell et al. 2022), emphasising the potential health impact of this landscape. At the same time, Accra experiences the triple burden of malnutrition, characterised by high rates of overweight and obesity alongside undernutrition and persistent micronutrient deficiencies, such as anaemia in women of reproductive age (Ghana Statistical Service 2015; Wrottesley et al. 2023). The prevalence of overweight is around 47% in children aged 6–16 years (Ganle, Boakye, and Baatiema 2019) and 40% in women of reproductive age (Ghana Statistical Service 2015).

Previous studies of diets in and around schools in Ghana included analysis of school meals provided through feeding programmes (Fernandes et al. 2017), highlighting non‐receipt of school meals as a predictor of bringing money to school to buy food, or focus on eating patterns of school children (Abizari and Ali 2019; Ogum‐Alangea et al. 2020), but neither provide information on the relationship of individual characteristics (such as amount of money brought) and diet quality. While existing literature from Ghana also shows that individuals are exposed to a large variety of economic and behavioural factors that influence their diet quality (Holdsworth et al. 2020; Laar et al. 2023), the pathways and association between these are still unclear. In their review of industrial diets among adolescents in Ghana, Sambu et al. (2024) found that most research on adolescents addresses unhealthy diets as an isolated behavioural or nutritional issue and fails to connect domains of the food system. Qualitative studies conducted among adolescents in Ghana underscore the pivotal role of the physical food environment, economic constraints, and cognitive factors such as food preferences in shaping dietary decisions (Holdsworth et al. 2020; Liguori et al. 2022; Mockshell et al. 2022; Pradeilles et al. 2021), which supports the need for quantitative research in this domain. In summary, existing research on socioeconomic drivers of dietary intake tends to either focus on adults (Karanja et al. 2022; Kushitor et al. 2023; Weil et al. 2023), include limited drivers on adolescent diet quality (Abizari and Ali 2019; Fernandes et al. 2017; Ganle, Boakye, and Baatiema 2019; Madzorera et al. 2023; Sambu et al. 2024) or utilise qualitative methods (Iyassu et al. 2023; Liguori et al. 2022) and does not reflect the unique situation of adolescents (Karanja et al. 2022; Kushitor et al. 2023). It is particularly important to better understand adolescent diets given the role healthy diets play for critical physiological and neurocognitive development during adolescence and the elevated burden of nutrition in Ghana overall.

We address this research gap by tracking the relationship between individual and behavioural factors in adolescents and the outcome of diet quality, measured by diet scores. In particular, we investigate the influence of five domains of individual factors, namely economic, cognitive, aspirational and situational factors as well as consumer behaviour on diet intake of school‐age adolescents in Accra. Thereby, this study aims to provide a comprehensive understanding of the factors associated with the food choices and eating patterns of school‐going adolescents in Accra.

## 2 Methods and Materials

### 2.1 Study Design and Population

Cross‐sectional in design, we used a two‐stage proportional stratified random sampling strategy. Three areas within Greater Accra were identified as sample sites based on similar economic, infrastructure and public health indicators reported in the 2021 census (Ghana Statistical Service 2022): Ayawaso East Municipality, Ablekuma Central Municipality and Accra Metro. Within these areas, eight public and four private schools were randomly selected (serving as the Primary Sampling Unit), following the public/private ratio and representative of official enrolment statistics. We selected 12 out of 115 Junior High Schools (JHS) and determined a minimum sample of 379 students (Secondary Sampling Unit), using a 5% margin of error, 95% confidence level and 50% sample proportion on an estimated 24,000 students in the identified metropolitan areas. In each of the identified schools, 36 students from grades JHS1 and JHS2 who attended school on the preceding day (to match the recall timeframe) were randomly selected, except for one school, where only 34 students were enroled in both grades, yielding a total of 428 students. After data cleaning and plausibility checks, 19 students were excluded due to implausible consumption patterns.

JHSs, which are mostly day schools in Ghana, do not provide institutional school meals (as basic schools) or financial support mechanisms as would be found, for example, in Senior High Schools (Free Senior High School Policy). Foods consumed by JHS students during a school day are therefore either purchased by the students, brought from home or potentially gifted. Indicators used in this study are aligned with the High Level Panel of Experts (HLPE) food systems framework (HLPE 2017) adapted for tracking global progress on the food systems dashboard (Fanzo et al. 2020). They are categorised according to five areas of individual and behavioural factors that influence diet choice: economic, cognitive, aspirational, situational and consumer behaviour (detailed mapping of the indicators to each factor is provided in Supporting Information: Material S1).

### 2.2 Protocol

We obtained written informed consent from all study participants: parents or legal guardians of all eligible students before their selection (*n* = 1510, response rate of parents > 80%) and written assent from the students before conducting the survey. All eligible and randomly selected students (‘participants’ from here on) assented to and completed the survey.

### 2.3 Data Collection

Participant data were collected in schools between the 7th and 28th of November 2022. This survey utilised five groups of variables collected as part of the questionnaire, which was pilot tested among 16 junior high students and conducted as described below (full survey questionnaires are reported in Supporting Information: Materials S2). Participant surveys were carried out Tuesdays to Fridays, so the dietary recall period (i.e., the previous day) would always refer to a school day.

#### 2.3.1 Anthropometric Variables

Anthropometric variables were collected as background information (not used in models) from participants, while dressed in school uniforms, without shoes. Height was measured using Leicester portable height measure (Marsden HM‐250P, 1 mm graduations) and weight was measured using portable weighing scales (Ingco HESA 41802, 0.1 kg precision). Given that students wear standardised school uniforms, weighing approximately 550 g, which was subtracted from measured weight, we do not expect bias from this in our anthropometric measurements (Censi et al. 2013).

#### 2.3.2 Knowledge, Attitude and Practice Scores

A formative set of 28 Knowledge (8), Attitude (8) and Practice (12) (KAP) questions were collected from students to capture individuals’ characteristics. Questions were based on established and validated protocols (Fautsch Macías, Glasauer, and Food and Agriculture Organization of the United Nations 2014; Moitra, Verma, and Madan 2021) and checked for face validity and applicability for the local context. Two researchers reviewed a set of 36 questions. Their feedback was integrated into the questionnaire and pilot tested with 16 students. After pilot testing, eight questions were removed from the survey. An overview of question per category and subcomponent is provided in Appendix Table A1. The maximum overall KAP Score that could be achieved was 36, with each of the individual components scored from 0 to 12. For knowledge and attitude, the individual subcategories were scored from 0 to 4, and for practices, the individual subcategories were scored from 0 to 3.

#### 2.3.3 Sociodemographic and Food Budget Information

Participants were asked to estimate their food budget for the previous 24 h. Specifically, we inquired how much money they had at their disposal for breakfast, lunch, snacks and total budget, in case food purchases occur outside of breakfast, lunch or snacks. Participants reported if they brought food from home for any of these eating occasions. They were additionally asked about other household characteristics, including maternal education, religion, ethnicity, maternal marital status and whether they live with their parents or a caretaker.

#### 2.3.4 Wealth Proxies

A collapsed 11‐item asset index was collected using a validated questionnaire from the equity tool project (Chakraborty et al. 2016). It was designed to capture the availability of key assets and assign scores, identifying the equivalent national wealth quintile, for each individual, based on cutoffs from national household surveys. Based on Maternal Health Survey 2017, urban‐specific cutoffs for wealth quintiles are available and were used to assign individuals to the appropriate quintiles.

#### 2.3.5 Diet Quality Questionnaire (DQQ)

A Ghana‐specific DQQ was used following established protocols (Herforth et al. 2020; Uyar et al. 2023). The DQQ recalls consumption of 29 food groups during the preceding 24 h, that is, whether a food group was consumed (binary). A detailed description of this methodology can be found elsewhere, as well as its applicability for adolescents (Herforth et al. 2020; Uyar et al. 2023; Wang et al. 2022). DQQ allows calculating different indicators related to dietary intake. For this study, we focused on the *Food Group Diversity Score* (FGDS; 0–10), *Global Dietary Recommendations (GDR) Score* (0–18), *Non‐Communicable Disease* (NCD) *Risk Score* (0–9) and *NCD‐Protect Score* (0–9) (Herforth et al. 2020). GDR Score is a composite of NCD‐Risk and NCD‐Protect (calculated as 9 + NCD‐Protect Score – NCD‐Risk Score), but for ease of reference, we reported results for all three variables.

#### 2.3.6 Definition of Outcome Variables

Outcome variables of interest in this study were FGDS, GDR Score, NCD‐Risk Score and NCD‐Protect Score. To capture trade‐offs between these linear scores, we defined categorical outcomes for our study cohort based on NCD‐Protect and NCD‐Risk Scores. These nutrition types were defined as: ‘Nutrition Struggler’ as the group that lacks diversity throughout (below 5 NCD‐Risk foods and below 5 NCD‐Protect foods). We defined ‘Nutrition Champions’ as our positive deviants: those who consumed 5 or more NCD‐Protect foods and 5 or less NCD‐Risk foods. ‘Nutrition Challengers’ were defined as negative deviants: those who consumed 5 or more NCD‐Risk foods while consuming 5 or less NCD‐Protect foods. Cutoffs for NCD‐Protect were set at 5 so positive deviants exceed the minimum threshold for dietary diversity (Arimond et al. 2021). Cutoffs for NCD‐Risk were also set at 5 to parallel NCD‐Protect cutoffs. More formally, this can be expressed as:

NutritionChallenger→NCD‐Risk≥5∩NCD‐Protect≤5∩NCD‐Risk>NCD‐Protect

NutritionChampion→NCD‐Protect≥5∩NCD‐Risk≤5∩NCD‐Protect>NCD‐Risk

NutritionStruggler→NCD‐Protect<5∩NCD‐Risk<5.

### 2.4 Definition of Predictors

Predictors used in this study were individuals' budgets measured in Ghanian Cedi (GHS) per day, as well as Knowledge, Attitude and Practice (KAP) scores. KAP scores were used in two different ways: (1) as aggregate scores for knowledge, attitude and practice, respectively, and (2) as expanded scores for all 10 Knowledge, attitude and practice subcategories (cf. previous section).

### 2.5 Assessment of Covariates

In this study, we assessed potential confounding variables informed by the conceptual work of Fanzo et al. (2020), also cf. Supporting Information: Materials S1, including sex, age, ethnicity, religion and a school dummy. Household characteristics included maternal education, maternal marital status, household wealth quintile and whether the child resided with their parents or someone else.

### 2.6 Statistical Analysis

Baseline characteristics of the study population were summarised as mean and standard deviation, disaggregated by sex to identify potential differences between sexes. To estimate the relationship between predictors and outcomes, we fitted the following multivariable linear regression models for each outcome:

(1) $$\begin{array}{lll} DQ_{i} & = & \beta 1\;*\;{\mathrm{DailyBudget}}_{i}+\beta 2\;*\;{\mathrm{Knowledge}}_{i}\\ & & +\;\beta 3\;*\;{\mathrm{Attitude}}_{i}+\beta 4\;*\;{\mathrm{Practice}}_{i}+\beta 5\;*\;X_{i}+\varepsilon_{i} \end{array}$$

where *DQ*_i_ represents different measures of diet quality, namely GDR Score, FGDS, NCD Protect or NCD Risk. The vector *X*_i_ contains socioeconomic background characteristics, namely sex, age, school, household wealth, religion, ethnicity, maternal education, maternal marital status and residence with parents.

We ran the model separately for each measure of diet quality *DQ*_i_. For a more nuanced analysis of the role of the budget and of the KAP scores, we ran a second model (expanded). In this model, *DailyBudget*_*i*_ is a vector of the daily budget consisting of breakfast, lunch and snack budget, *Knowledge*_*i*_ is the vector of three subcomponent knowledge scores, *Attitude*_*i*_ is the vector of three subcomponent Attitude Scores, *Practice*_*i*_ is the vector of the three subcomponent Practice Scores and *X*_i_ is the vector of socioeconomic background characteristics, namely sex, age, school, household wealth, religion, ethnicity, maternal education, maternal marital status and residence with parent. We adjusted for multiple hypothesis testing using False Discovery Rate set at 0.05 following the Benjamini‐Hochberg procedure (Benjamini and Hochberg 1995) and reported both p‐values and q‐values.

We also applied a multinomial logistic regression model on nutrition type and the previously described predictors:

(2) $$\begin{array}{ccl} NT_{i} & = & \gamma 1\;*\;{\mathrm{DailyBudget}}_{i}+\gamma 2\;*\;{\mathrm{Knowledge}}_{i}\\ & & +\;\gamma 3\;*\;{\mathrm{Attitude}}_{i}+\gamma 4\;*\;{\mathrm{Practice}}_{i}+\gamma 5\;*\;X_{i}+\varepsilon_{i} \end{array}$$

where:

*NT*_i_ (Nutrition Type) represents three categories (Nutrition Champion, Nutrition Challenger and Nutrition Struggler), and *X*_i_ is the vector of socioeconomic background characteristics: sex, age, school, household wealth, religion, ethnicity, maternal education, maternal marital status and residence with parents. We set the Nutrition Champions as the reference group.

## 3 Results

### 3.1 Basic Characteristics of Study Population

Table 1 reports the characteristics of the study participants according to sex. The median age for all study participants was 14 years. Half of the participants were female. Nearly all (98%) of the students reported having purchased some foods with the budget available to them.

**Table 1:** Summary Characteristics of study population, by sex.

	All (*n* = 409)		Female (*n* = 206, 50.4%)		Male (*n* = 203, 49.6%)	
Variable	Mean	SD	Mean	SD	Mean	SD
Age (in years)	14.35	(1.28)	14.27	(1.13)	14.42	(1.41)
Body mass index (BMI)	19.70	(3.70)	20.73	(4.05)	18.65	(2.97)
BMI for age (z‐score)	−0.18	(1.23)	0.13	(1.20)	−0.49	(1.19)
GDR score (0–18)	9.15	(2.07)	9.16	(2.10)	9.14	(2.04)
NCD‐Protect score (0–9)	3.24	(1.67)	3.39	(1.73)	3.08	(1.61)
NCD‐Risk score (0–9)	3.09	(2.01)	3.23	(2.04)	2.94	(1.96)
FGD score (0–10)	5.12	(1.63)	5.19	(1.62)	5.05	(1.63)
Achieved MDD‐W (%)	N/A		65.0	(0.48)	N/A	
Budget total (GHS)	10.20	(5.42)	9.86	(5.05)	10.54	(5.76)
Budget breakfast (GHS)	2.80	(2.84)	2.68	(2.71)	2.92	(2.97)
Budget lunch (GHS)	4.24	(2.86)	4.15	(2.79)	4.33	(2.93)
Budget snacks (GHS)	2.24	(2.51)	2.21	(2.51)	2.27	(2.53)
Knowledge score (0–12)	6.36	(1.99)	6.40	(1.95)	6.31	(2.03)
Attitude score (0–12)	8.17	(1.30)	8.24	(1.37)	8.10	(1.22)
Practice score (0–12)	5.11	(1.63)	5.12	(1.71)	5.09	(1.54)

*Note:* All numbers are mean values, and parentheses show standard deviations (SD). 1 GHS ≈ 0.07 USD (2022).

Abbreviations: BMI, Body Mass Index; FGD, Food Group Diversity; GDR, Global Dietary Recommendation; GHS, Ghana Cedis; MDD‐W, Minimum Dietary Diversity for Women; NCD, Non‐Communicable Diseases.

### 3.2 Drivers of Diet Quality Scores

Table 2 reports the results of the multivariable linear regression model. We first considered students' budget. Adjusting for all other factors, students' total budget was positively associated with a more diverse diet as measured by the FGDS (*β* = 0.12, 95% confidence interval [CI] 0.09–0.15). A higher budget was associated with a higher consumption of NCD‐Protect foods (*β* = 0.10, 95% CI 0.06–0.13). However, a higher budget was also associated with higher consumption of NCD‐Risk foods (*β* = 0.16, 95% CI 0.13–0.20). Since the total budget had a stronger association with the consumption of NCD‐Risk foods compared to NCD‐Protect foods, the total budget was associated with an overall decrease in diet quality as measured by the GDR Score (*β* = −0.07, 95% CI −0.11 to −0.03). Turning towards non‐economic drivers of diet quality, we found that, while adjusting for other variables, both the attitude towards and practices of healthy eating are positively associated with the GDR Score (attitude *β* = 0.23, 95% CI 0.07–0.39; practices *β* = 0.17, 95% CI 0.04–0.30). Knowledge about healthy eating, on the other side, was negatively associated with FGDS and NCD‐Protect (*β* = −0.12, 95% CI −0.19 to −0.04 and *β* = −0.10, 95% CI −0.18, −0.02), while practices were positively associated with these two outcomes (*β* = 0.20, 95% CI 0.11–0.29 and *β* = 0.24, 95% CI 0.14–0.34). Finally, attitude towards healthy eating was negatively associated with the consumption of NCD‐Risk foods (*β* = −0.17, 95% CI −0.31 to −0.03). Only the associations between knowledge and NCD‐Protect as well as attitude and NCD‐Risk were not robust when accounting for multiple hypotheses testing.

**Table 2:** Results of multiple linear regression.

	GDR score			FGDS			NCD protect			NCD risk		
	Estimate			Estimate			Estimate			Estimate		
Predictors	(95% CI)	*p*‐value	*q*‐value	(95% CI)	*p*‐value	*q*‐value	(95% CI)	*p*‐value	*q*‐value	(95% CI)	*p*‐value	*q*‐value
Total budget	−0.07*	**0.001**	**0.024**	0.12***	**< 0.001**	**< 0.001**	0.10***	**< 0.001**	**< 0.001**	0.16***	**< 0.001**	**< 0.001**
	(−0.11 to −0.03)			(0.09–0.15)			(0.06–0.13)			(0.13– 0.20)		
Knowledge score	−0.04	0.447	0.661	−0.12*	**0.003**	**0.026**	−0.1	**0.014**	0.061	−0.06	0.201	0.621
	(−0.15 to 0.06)			(−0.19 to −0.04)			(−0.18 to −0.02)			(−0.16 to 0.03)		
Attitude score	0.23*	**0.005**	**0.043**	0.01	0.813	0.959	0.06	0.345	0.554	−0.17	**0.021**	0.145
	(0.07–0.39)			(−0.10 to 0.13)			(−0.06 to 0.18)			(−0.31 to −0.03)		
Practice score	0.17	**0.01**	0.067	0.20***	**< 0.001**	**< 0.001**	0.24***	**< 0.001**	**< 0.001**	0.07	0.261	0.635
	(0.04–0.30)			(0.11–0.29)			(0.14–0.34)			(−0.05 to 0.18)		
Observations	409			409			409			409		
*R* ^2^/*R* ^2^ adjusted	0.163/0.089			0.283/0.220			0.227/0.159			0.282/0.218		

*Note:p*, *p*‐value; *q*‐value, FDR adjusted *p*‐value using the Benjamini–Hochberg procedure. Models were adjusted for age, sex, wealth quintile, religion, ethnicity, maternal education, marital status of the mother, residency of the child (e.g., living with parents or caretaker) and school.

Abbreviations: FGDS, Food Group Diversity Score; GDR, Global Dietary Recommendations; KAP, Knowledge, Attitude and Practices; NCD, non‐communicable disease.

* *p* < 0.05

** *p* < 0.01

*** *p* < 0.001.

Table 3 reports expanded results of the multivariable linear regression model, which showed that budget dedicated to snacks was inversely associated with GDR Score (GDR: *β* = −0.14, 95% CI −0.22 to −0.05) and positively associated with FGDS, NCD‐Protect and NCD‐Risk (FGDS: *β* = 0.15, 95% 0.09–0.21, NCD‐Protect: *β* = 0.13, 95% 0.06–0.19, NCD‐Risk: *β* = 0.26, 95% 0.19–0.34). Breakfast and lunch budgets were positively associated with FGDS, NCD‐Protect and NCD‐Risk but showed no association with GDR. Knowledge of NCDs was inversely associated with NCD‐Protect (*β* = −0.38, 95% CI −0.63 to −0.13). Attitude towards the benefits of healthy eating showed positive associations with GDR Score (*β* = 0.59, 95% CI 0.20–0.98), as did family practice scores (*β* = 0.53, 95% CI 0.20–0.86). Readiness to change was positively associated with FGDS (*β* = 0.28, 95% CI 0.09–0.47) and NCD‐Protect (*β* = 0.37, 95% CI 0.17–0.57).

**Table 3:** Regression results of the expanded multivariable linear regression models.

	GDR score			FGDS			NCD protect			NCD risk		
	Estimate			Estimate			Estimate			Estimate		
Predictors	(95% CI)	*p*‐value	*q*‐value	(95% CI)	*p*‐value	*q*‐value	(95% CI)	*p*‐value	*q*‐value	(95% CI)	*p*‐value	*q*‐value
Breakfast budget	−0.01	0.769	0.865	0.12***	**< 0.001**	**< 0.001**	0.09*	**0.003**	**0.023**	0.10*	**0.004**	**0.033**
	(−0.08 to 0.06)			(0.06–0.17)			(0.03–0.14)			(0.03–0.16)		
Lunch budget	0.02	0.683	0.819	0.12***	**< 0.001**	**0.001**	0.13**	**< 0.001**	**0.001**	0.12*	**0.002**	**0.016**
	(−0.06 to 0.10)			(0.06–0.18)			(0.07–0.19)			(0.04–0.19)		
Snacks budget	−0.14*	**0.002**	**0.02**	0.15***	**< 0.001**	**< 0.001**	0.13**	**< 0.001**	**0.003**	0.26***	**< 0.001**	**< 0.001**
	(−0.22 to −0.05)			(0.09–0.21)			(0.06–0.19)			(0.19–0.34)		
Knowledge—Eating habits	−0.35	0.095	0.283	−0.21	0.181	0.543	−0.26	0.101	0.266	0.08	0.66	0.866
	(−0.75 to 0.06)			(−0.51 to 0.10)			(−0.58 to 0.05)			(−0.28 to 0.45)		
Knowledge—NCDs	−0.2	0.228	0.478	−0.31	**0.011**	0.076	−0.38*	**0.003**	**0.023**	−0.18	0.223	0.52
	(−0.52 to 0.12)			(−0.55 to −0.07)			(−0.63 to −0.13)			(−0.47 to 0.11)		
Knowledge—Food groups	0.25	0.165	0.384	0.03	0.848	0.977	0.22	0.116	0.287	−0.03	0.855	0.921
	(−0.10 to 0.59)			(−0.23 to 0.28)			(−0.05 to 0.49)			(−0.34 to 0.28)		
Attitude—Benefits	0.59*	**0.004**	**0.03**	0.01	0.925	0.977	0.1	0.501	0.702	−0.48	**0.008**	0.054
	(0.19 to 0.98)			(−0.28 to 0.31)			(−0.20 to 0.41)			(−0.84 to −0.13)		
Attitude—Barriers	0.16	0.305	0.53	−0.03	0.777	0.977	0.06	0.626	0.796	−0.1	0.474	0.82
	(−0.15 to 0.47)			(−0.26 to 0.20)			(−0.18 to 0.30)			(−0.38 to 0.18)		
Attitude—Susceptibility	0.16	0.131	0.345	0	0.999	0.999	0.03	0.754	0.905	−0.13	0.16	0.431
	(−0.05 to 0.37)			(−0.16 to 0.16)			(−0.14 to 0.19)			(−0.32 to 0.05)		
Practices—Eating	0.05	0.782	0.865	−0.03	0.824	0.977	−0.05	0.738	0.905	−0.11	0.551	0.864
	(−0.33 to 0.44)			(−0.32 to 0.26)			(−0.35 to 0.25)			(−0.46 to 0.24)		
Practices—Readiness	0.15	0.257	0.491	0.28*	**0.004**	**0.032**	0.37**	**< 0.001**	**0.005**	0.22	0.063	0.265
	(−0.11 to 0.40)			(0.09–0.47)			(0.17–0.57)			(−0.01 to 0.45)		
Practices—Peers	−0.12	0.586	0.745	0.34	**0.031**	0.161	0.29	0.084	0.247	0.4	**0.036**	0.188
	(−0.54 to 0.30)			(0.03–0.66)			(−0.04 to 0.62)			(0.03–0.78)		
Practices—Family	0.53*	**0.002**	**0.02**	0.22	0.077	0.293	0.29	**0.027**	0.115	−0.24	0.122	0.427
	(0.20–0.86)			(−0.02 to 0.47)			(0.03–0.55)			(−0.54 to 0.06)		
Observations	409			409			409			409		
*R* ^2^/*R* ^2^ adjusted	0.202/0.113			0.286/0.206			0.260/0.177			0.311/0.234		

*Note: p*, *p*‐value; *q*‐value, FDR adjusted *p*‐value using the Benjamini–Hochberg procedure. Models were adjusted for age, sex, wealth quintile, religion, ethnicity, maternal education, marital status of the mother, residency of the child (e.g., living with parents or caretaker) and school.

Abbreviations: FGDS, Food Group Diversity Score; GDR, Global Dietary Recommendations; KAP, Knowledge, Attitude and Practices; NCD, non‐communicable disease.

* *p* < 0.05

** *p* < 0.01

*** *p* < 0.001.

### 3.3 Drivers of Nutrition Types

In the next step, we assigned students to the three nutrition types. We found that almost two thirds (66%) of study participants belonged to the ‘Struggler’ group. Around 16% belonged to the ‘Challenger’ and 13% belonged to the ‘Champion’ group. About 5% were not assigned to any nutrition type.

Correlates of group assignment based on logistic regression models are shown in Table 4. Comparing Strugglers and Challengers to Champions, we found that a one‐unit (GHS) increase in total budget was associated with lower relative odds of being a Struggler as compared to being a Champion (OR 0.88; 95% CI 0.82–0.95). A one‐unit increase in attitude score was associated with lower relative odds of being a Challenger as compared to being a Champion (OR 0.63; 95% CI 0.45–0.90). A one‐unit increase in practice score was associated with lower relative odds of being a Struggler as compared to being a Champion (OR 0.66; 95% CI 0.53–0.83).

**Table 4:** Regression results for multinomial logistic regression models, ref = Champions (positive deviants).

	Challenger		Struggler	
Predictors	Odds ratios (95% CI)	*p*‐value	Odds ratios (95% CI)	*p*‐value
Total budget	1.07 (0.98–1.16)	0.145	0.88** (0.82–0.95)	0.001
Knowledge score	1.06 (0.85–1.32)	0.604	1.08 (0.90–1.28)	0.418
Attitude score	0.63* (0.45–0.90)	0.011	0.84 (0.64–1.12)	0.240
Practices score	0.76 (0.58–1.00)	0.050	0.66*** (0.53–0.83)	< 0.001
Observations	385			
*R* ^2^/*R* ^2^ adjusted	0.192/0.189			

*Note:* Parentheses show 95% confidence intervals (CI); *p*, *p*‐value. Models were adjusted for age, sex, wealth quintile, religion, ethnicity, maternal education, marital status of the mother, residency of the child (e.g., living with parents or caretaker) and school.

* *p* < 0.05

** *p* < 0.01

*** *p* < 0.001.

For the expanded model, being in the Struggler group was inversely associated with the breakfast budget (OR: 0.85; 95% CI 0.74–0.97). The only difference between Challengers and Champions (Reference) was lower attitude towards the benefits of eating healthy (OR = 0.41; 95% CI −0.12 to 0.99) and family practices (OR = 0.48; 95% CI 0.23–1.00) (Table 5).

**Table 5:** Regression results for multinomial logistic regression models, ref = Champions (positive deviants).

	Challenger		Struggler	
Predictors	Odds ratios (95% CI)	*p*‐value	Odds ratios (95% CI)	*p*‐value
Breakfast budget	1.01 (0.86–1.17)	0.948	0.85* (0.74–0.97)	0.013
Lunch budget	1.11 (0.93–1.34)	0.241	0.90 (0.77–1.04)	0.153
Snacks budget	1.13 (0.95–1.34)	0.159	0.90 (0.77–1.04)	0.150
Knowledge—Eating habits	0.95 (0.40–2.24)	0.912	0.87 (0.43–1.75)	0.695
Knowledge—NCDs	1.96 (0.95–4.05)	0.068	2.19* (1.21–3.98)	0.010
Knowledge—Food groups	0.79 (0.39–1.61)	0.518	0.74 (0.42–1.31)	0.298
Attitude—Benefits	0.41* (0.17–0.99)	0.047	0.57 (0.28–1.16)	0.120
Attitude—Barriers	0.64 (0.32–1.26)	0.196	0.70 (0.40–1.20)	0.193
Attitude—Susceptibility	0.79 (0.50–1.25)	0.319	1.18 (0.81–1.72)	0.383
Practices—Eating	0.76 (0.32–1.78)	0.524	0.71 (0.37–1.39)	0.320
Practices—Readiness	0.76 (0.43–1.33)	0.338	0.59* (0.37–0.94)	0.027
Practices—Peers	1.23 (0.51–2.99)	0.647	0.67 (0.32–1.40)	0.281
Practices—Family	0.48* (0.23–1.00)	0.049	0.62 (0.33–1.14)	0.122
Observations	385			
*R* ^2^/*R* ^2^ adjusted	0.216/0.213			

*Note:* Parentheses show 95% confidence intervals (CI); *p*, *p*‐value. Models were adjusted for age, sex, wealth quintile, religion, ethnicity, maternal education, marital status of the mother, residency of the child (e.g., living with parents or caretaker) and school.

Abbreviations: FGDS, Food Group Diversity Score; GDR, Global Dietary Recommendations; KAP, Knowledge, Attitude and Practices; NCD, non‐communicable disease.

* *p* < 0.05

** *p* < 0.01

*** *p* < 0.001.

## 4 Discussion

In our exploratory study on the influence of five domains of individual factors (economic, cognitive, aspirational, situational and consumer behaviour) on the diet intake of school‐age adolescents in Accra, we report three key results.

First, a higher budget alone, keeping all other factors fixed, was associated with slightly more unfavourable food groups in the diet. This means that individuals with a higher budget tended to consume more NCD‐Risk foods (e.g., sugar‐sweetened beverages, sweets, processed meat or packaged, ultra‐processed salty snacks) than NCD‐Protect foods (e.g., whole grains, pulses, dark green leafy vegetables). This complements the established relationship at the household level between diet quality and food expenditure (Pechey and Monsivais 2016), income (Bouis, Eozenou, and Rahman 2011; Colen et al. 2018; French et al. 2019; Muhammad et al. 2017) or socioeconomic status (Cutler et al. 2011; Fitzgerald et al. 2013). Larger food budget was associated with higher intake of favourable group, but also unfavourable food groups, such as processed meat, sweets or packaged, salty snacks. Evidence on this well‐documented phenomenon (Global Alliance for Improved Nutrition GAIN, 2020; Monteiro et al. 2013; Ogum‐Alangea et al. 2020; Westbury et al. 2021), observed particularly alongside a growing urban middle‐class in low‐ and middle‐income countries (Colozza, 2022; Karanja et al. 2022; Landais et al. 2023; Li et al. 2020), parallels increased attention to indicators reflecting healthy and unhealthy elements of diets (Herforth et al. 2020). Our results suggest that for adolescents within the urban context, higher consumption of NCD‐Risk foods was predominantly driven by snack budget, that is, having cash available to spend on food outside of specific meals.

Second, beyond food budget, the results show considerable differences between each of the two main pathways of the Healthy Diet recommended by the World Health Organization—*increasing* favourable food groups and *moderating* unfavourable food groups. A higher attitude score on nutrition (considering oneself susceptible to nutrition‐related diseases and perceiving benefits from adequate dietary intake) was negatively associated with the consumption of NCD‐Risk foods. Higher practice scores, in particular family practices and practices that reveal the readiness to change, were positively associated with NCD‐Protect foods. Previous studies on obesity also show that attitudes and perceptions about risk factors for being overweight are associated with the prevalence of overweight (Bean et al. 2018; Manggabarani et al. 2020; Oyewande et al. 2019), and research on psycho‐social and cognitive determinants, such as knowledge, attitudes and practices, has established that these traits can strongly influence dietary intake. But while cognitions—including risk perception, preferences, food characteristics or health and nutrition knowledge—are considered important determinants of individuals' diets (Karanja et al. 2022; Manggabarani et al. 2020; Patrick and Nicklas 2005; Peters et al. 2009; Shahi et al. 2023), the role of factual knowledge or pure competence in nutrition itself is unclear: Several studies from predominantly high‐income countries show promising results of improved dietary habits after nutrition counselling or knowledge intervention (Amoore et al. 2023; Brennan et al. 2021; Kullen et al. 2022), but others find that knowledge in nutrition by itself is insufficient to improve diets (Campbell et al. 2013; Tabbakh and Freeland‐Graves 2016). The association of beneficial family practices with improved dietary practices is supported by evidence from high‐income‐countries (Gillman, 2000; Neumark‐Sztainer et al. 2008; Nicklas et al. 2001; Patrick and Nicklas 2005) and low‐middle‐income countries (Glanz et al. 2021; Sedibe et al. 2018; Sirasa et al. 2019). The role of peer practices was less pronounced, both in this study and in the literature. Conceptually, the food intake of adolescents is related to peer values or social norms (Contento et al. 2006; Stok et al. 2016). While empirical, qualitative studies on this subject highlight the importance of peer norms generally, they emphasise the budgetary constraint to be more dominant (Holdsworth et al. 2020; Liguori et al. 2022).

Of note is the reported negative association of knowledge of NCDs and favourable food groups (FGDS and NCD‐Protect). Several factors could explain this counterintuitive finding: For one, individuals with lower scores in favourable food groups could be externally targeted by nutrition knowledge interventions. Additionally, individuals with lower scores in favourable food groups may also have to think about their food choices more thoroughly and regularly than those with high favourable food group intake, thereby increasing competency on nutrition. Finally, individuals with higher scores in favourable food groups could be 'ignorant' of some of the NCD‐related facts, because consuming less unfavourable foods may lead to less domain knowledge of diet‐related NCD risks.

Our third key finding is that Nutrition Champions—the positive deviants, who exhibited low intake of NCD‐Risk and relatively higher intake of NCD‐Protect food groups—did not have higher food budget than their counterparts (Nutrition Challengers) but differ in attitude and practice scores. These findings were driven by the higher sub‐components of attitudes towards benefits of healthy eating and family practices, respectively. A study from France using a positive deviance approach has documented higher nutritional quality at no additional cost within a low‐income group (Marty et al. 2015), suggesting that some improvement in diet quality can be achieved without increasing cost. Similar to a recent study from Nepal, which documented positive deviants (with a higher dietary diversity score) to be more engaged in joint family practices such as preparing meals (Shahi et al. 2023), we also found that positive deviants have higher family practice scores. Notably, the number of positive deviants was fairly low (13%), indicating that only a few students managed to strike a favourable balance in their food group intake.

This study has several strengths: We based our findings on a quantified analysis of both economic and behavioural drivers and used an extensive set of covariates (behavioural and socioeconomic). We also included dietary intake measurements for both favourable and unfavourable dietary patterns and focused on JHS students in Accra, who (have to) purchase much of their own food. However, despite these strengths, some limitations remain. The data on dietary intake, background characteristics and KAP details were all based on self‐reported measurements, which could introduce some bias. Second, dietary intake was collected based on the DQQ methodology. This means that frequency dietary intake was only collected at the food group level and may not be analysed beyond that. Furthermore, we also did not collect any information on portion size, which may explain some variation in diet quality. However, existing evidence from other countries indicates that portion size adds limited information in explaining variation in diet quality (Nöthlings et al. 2003). As the suite of indicators derived from the DQQ do not require portion size, we consider it to be sufficiently granular enough for the purpose of our study. Finally, despite the number of covariates included in the study, some residual confounding could still occur.

The findings of this study coherently spell out a need for double‐duty actions in Accra and comparable urban areas in sub‐Saharan Africa: JHS students in Accra typically carry money with them and spend it mainly (both by majority of students and by majority of money spent) on foods that are considered risk factors for NCDs. At the same time, nearly one‐third of the girls in the study cohort do not consume a diet that is minimally diverse (MDD‐W) and will likely not meet requirements for micronutrient density.

Policy interventions could regulate foods sold and consumed on school grounds during school time, which falls under the mandate of the school health authorities (School Health Education Programme). Another opportunity lies in the current national effort to develop four double‐duty food‐based policy bundles – comprising a front‐of‐pack warning label, marketing restrictions, public food procurement and ultra‐processed food taxation (Laar et al. 2023). Public food procurement (done by, or with oversight from government and other public sector agencies) in programmes such as school meals can help in creating a healthy school food environment, especially when they are aligned with Food Based Dietary Guidelines (Ministry of Food and Agriculture, & University of Ghana School of Public Health, 2023). Targeted subsidies and taxes for foods sold at schools could encourage a more favourable diversity in consumption. Identifying the best intervention and policy design may require localised research and testing different approaches. More research needs to be directed towards the decision making around food purchases of adolescents in their specific food environment (Pradeilles et al. 2021), to leverage the opportunity this age group has for public health over the course of their lifetime.

## 5 Conclusion

Higher budget for food was associated with food group diversity but not with improved diet quality. Favourable and unfavourable dietary patterns were associated with different drivers. While beneficial practices were linked to increased intake of favourable food groups, positive attitudes towards nutrition are linked to moderation of unfavourable food groups. With these dynamics, it is paramount for policy makers and programme implementers to pursue double‐duty actions regarding healthy diets. This means to ensure that favourable foods are more readily available, accessible and affordable for consumption while supporting moderation of unfavourable foods. Given the critical role that adolescents play in public health, ensuring healthy options tailored to their own budget are available should be the first step to improve their dietary intake.

## Author Contributions

Janosch Klemm, Christian Borgemeister and Ute Nöthlings designed the research study and contributed to protocol development. Janosch Klemm led the data collection and management. Janosch Klemm analysed the data with support from Samuel Muli. Samuel Muli, Kolade Oluwagbemigun, Martin Parlasca, Aba Crentsil, Deda Ogum and Ute Nöthlings contributed to research methodology and validation. Janosch Klemm, Samuel Muli, Kolade Oluwagbemigun and Martin Parlasca wrote the first draft of the paper. Aba Crentsil, Deda Ogum, Peter Quartey, Amos Laar, Anna Lartey, Christian Borgemeister and Ute Nöthlings reviewed and critically revised the paper. All authors have read and approved the final manuscript.

## Ethics Statement

Ethical approval for the study was received from the Ghana Health Service Ethics Review Committee (GHS‐ERC 021/08/22), the University of Ghana, Legon (ECH 064/22‐23), and the Center of Development Research Ethics Committee (15c_22).

## Consent

Written consent of legal guardians was obtained for all participants under the age of 18. Written assent of all participants was obtained before being interviewed.

## Conflicts of Interest

The authors declare no conflicts of interest.

## Supporting information

Supporting information.

Supporting information.

## Data Availability

Data will be made available upon reasonable request. The data that support the findings of this study are available on request from the corresponding author. The data are not publicly available due to privacy or ethical restrictions.

## 1

**Table A1:** Questions and corresponding groups from the KAP survey (based on Moitra, Verma, and Madan 2021 and Fautsch Macías, Glasauer, and Food and Agriculture Organization of the United Nations 2014).

	Sub‐group	Questions
Knowledge (0–12)	Knowledge about NCDs (0–4)	– What are the health problems that can occur when a person is overweight or obese? – Can you tell me some reasons why people might be overweight or obese? – How can people prevent overweight and obesity?
	Knowledge about food groups (0–4)	– Which food groups should take the most space on your plate? – How many servings of fruit and vegetables should children consume per day? – Which foods should make up the smallest part of your diet?
	Knowledge about healthy eating (0–4)	– Some children do not have breakfast before going to school and are hungry in class. What is the consequence for children of not having breakfast and being hungry at school? – Why should parents discourage sticky and sugar‐rich foods, such as sweets and candies? (Why is it so bad to eat too many sweets and candies?) – What are healthy alternatives to Sugar‐Sweetened Beverages?
Attitude (0‐12)	Perceived susceptibility (0–4)	– I will get diseases if I don't eat healthily – I am worried about becoming Overweight/Obese – I am worried about getting Diabetes – I am worried about getting heart diseases
	Perceived benefits (0–4)	– Healthy Eating can reduce the risk of diseases – Eating fruits can help you fight infections – Eating vegetables can help you lose weight – Regular breakfast helps improve being alert
	Perceived barriers (0–4)	– It is difficult to eat two (2) fruits every day – I am not sure what and how much I should eat – I typically do not have enough money for eating fresh fruits – There are no healthy food options offered in and around school
Practices (0–12)	Readiness to change (0–3)	– I try to eat breakfast everyday – I try to eat two pieces of fruit everyday – I want to improve my eating habits – I would like to drink less soft drinks
	Peer habits (0–3)	– In the last 7 days, how often did you and your friends ‐ eat breakfast together away from home – In the last 7 days, how often did you and your friends ‐ eat a mid‐morning snack together – In the last 7 days, how often did you and your friends ‐ eat lunch together away from home/at or around school – In the last 7 days, how often did you and your friends ‐ eat dinner together away from home
	Family habits (0–3)	– In the last 7 days, how often did you and your FAMILY ‐ eat out at a restaurant/order take away? – In the last 7 days, how often did you and your FAMILY ‐ eat a home‐cooked evening meal together at home? – In the last 7 days, how often did you and your FAMILY ‐ eat breakfast together at home? – In the last 7 days, how often did you and your FAMILY ‐ eat lunch together at home/send you with packed lunch to school?
	Eating patterns (0–3)	– In a normal school week (5 days) how often do you have breakfast before leaving for school? – In a normal school week (5 days) how often do you bring your lunch to school? – In a normal school week (5 days) how often do you skip a meal?

Abbreviation: NCD, Non‐Communicable Diseases.
