# Supplementary material for: Individual‐Level Drivers of Food Choices and Diet Quality Among Adolescents in Urban West Africa: Evidence From Accra, Ghana
Source: Matern Child Nutr. 2024 Dec 9;21(2):e13775. doi: 10.1111/mcn.13775 (PMC11956040; doi:10.1111/mcn.13775)
Supplement: Supplementary file 1 — Supporting information. [file MCN-21-e13775-s001.pdf]

ALL_STUDENTS

Background

» Intro

Introduction

Please introduce yourself and make sure that the ASSENT FORM is signed by the students.

- OK

Have you been in school yesterday?

Only continue with the questionnaire if the student was in school yesterday.

- OK

Hello and Welcome. Thank you for participating in this study. Before we begin, I would like to remind you that this questionnaire is completely anonymous. Once you leave this conversation, no one, not even us, will be able to connect the answers to you. I would therefore like to ask you to answer to the best of your knowledge and truthfully. This will hopefully help us inform and guide decision making that will improve the food situation in and around schools.

- OK

Name of School

- [redacted]
- [redacted]
- [redacted]
- [redacted]
- [redacted]
- [redacted]
- [redacted]
- [redacted]
- [redacted]
- [redacted]
- [redacted]
- [redacted]

Respondent ID

Consists of School ID - Enumerator ID - # of Student of the day, e.g. 1-1-1

» Background I

Sex

- Male
- Female
- Prefer not to say

Age

Weight (in kg, e.g. 67.8 kg)

Height (in cm, e.g. 140.5 cm)

» Background II

Religion

- Christian
- Muslim
- Other

Ethnicity

- Ga/Dangme
- Akan (e.g. Ashanti, Fanti, etc)
- Ewe
- Northern (e.g. Bagomba, Frafa, etc)
- Other

» Background III

Do you live with...

- your mother or father
- a caregiver (aunt, uncle, grandmother/father)

What is the highest completed level of education of your mother/female caregiver?

- No formal education
- Basic School
- JHS
- Secondary (middle school, SHS, etc)
- Technical/Vocational
- University
- Other
- Don't know
- N/A

What is the marital status of your mother/female caregiver?

- married
- never been married
- divorced/separated
- cohabitating
- widowed
- N/A

Wealth Asset Index

I will now ask you about some of the things you have at home. Please answer YES or NO to each section.

- OK

» Household Assets

Q1: Does your household have a radio?

- YES
- NO

Q2: Does your household have a television?

- YES
- NO

Q3: Does your household have a computer/ tablet?

- YES
- NO

Q4: Does your household have a refrigerator?

- YES
- NO

Q5: Does your household have a cabinet/cupboard?

- YES
- NO

» Individual ownership

Q6: Does any member of your household own a wrist watch?

- YES
- NO

Q7: Does any member of your household have a bank account?

- YES
- NO

» Infrastructure and Sanitation

Q8: What is the main source of drinking water for members of your household?

- Sachet water (includes bottled water or dispenser)
- Other source of drinking water

Q9: What kind of toilet facility do members of your household usually use?

If they do not have it in their house, it is other toilet facility.

- Flush to manhole/ septic tank (not shared)
- Other toilet facility

Q10: What type of fuel does your household mainly use for cooking?

- Wood
- LPG (Gas)
- Other source of cooking fuel

Q11: What is the main material of the floor in your dwelling?

- Cement
- Other material

DQQ

READ THE FOLLOWING BEFORE MOVING TO THE QUESTIONS.

Now I'd like to ask you some yes-or-no questions about foods and drinks that you consumed yesterday during the day or night, whethe you had it at home or somewhere else. First, I would like you to think about yesterday, from the time you woke up through the night. Think to yourself about the first thing you ate or drank after you woke up in the morning ... Think about where you were when you had any food or drink in the middle of the day ... Think about where you were when you had any evening meal ... and any food or drink you may have had in the evening or late-night... and any other snacks or drinks you may have had between meals throughout the day or night. I am interested in whether you had the food items I will mention even if they were combined with other foods. Please listen to th list of foods and drinks, and if you ate or drank ANY ONE OF THEM, say yes.

- OK

» Yesterday, did you eat any of the following foods:

1 - Bread, rice, waakye, jollof, fried rice, omutuo, or rice porridge?

- YES
- NO

2 Kenkey, banku, tou zaafi, Hausa koko, akple, roasted maize, boiled maize, or tom brown?

- YES
- NO

3 Fufu, gari, kokonte, cassava, yam, cocoyam, plantain, or sweet potato?

- YES
- NO

4 Beans, or bambara beans?

- YES
- NO

» Yesterday, did you eat any of the following vegetables:

5 Carrots, or sweet potatoes that are orange inside?

- YES
- NO

6.1 Cocoyam leaves, amaranth leaves, ademe, ayoyo, potato leaves, cassava leaves, or bokoboko?

- YES
- NO

6.2 Baobab leaves, cowpea leaves, roselle leaves or bra, kenaf, African eggplant leaves, or West India nettle?

- YES
- NO

7.1 Tomatoes, okro, garden eggs, or cabbage?

- YES
- NO

7.2 Sweet green pepper, lettuce, cucumber, or mushrooms?

- YES
- NO

» Yesterday, did you eat any of the following fruits:

8 Mango, papaya, or African star apple?

- YES
- NO

9 Orange or tangerine?

- YES
- NO

10.1 Banana, pineapple, avocado pear, watermelon, apple, or guava?

- YES
- NO

10.2 Soursop, coconut, velvet tamarind, baobab, ebony fruit, or shea fruit?

- YES
- NO

» Yesterday, did you eat any of the following sweets:

11 Cakes, biscuits, rock bun, toogbee or bofrot?

- YES
- NO

12 Toffees, chocolates, ice cream, or FanYogo?

- YES
- NO

» Yesterday, did you eat any of the following foods of animal origin:

13 Eggs?

- YES
- NO

14 Cheese curds or wagashi?

- YES
- NO

15 Brukina or drink yogurt?

- YES
- NO

16 Sausages or corned beef?

- YES
- NO

17 Beef, goat, sheep, liver, or intestine?

- YES
- NO

18 Pork , grasscutter , rabbit, or bush meat?

- YES
- NO

19 Chicken, gizzard, or Guinea fowl?

- YES
- NO

20 Fish, dried fish, koobi, anchovies, smoked herring, crab, or shrimp?

- YES
- NO

» Yesterday, did you eat any of the following other foods:

21 Groundnuts, kuli kuli, groundnut paste, groundnut soup, agushi stew, neri soup, or cashews?

- YES
- NO

22 Packaged yellow plantain chips or potato chips or Pringles?

- YES
- NO

23 Indomie?

- YES
- NO

24 French fries, fried yam, fried potato, atomo, spring rolls, fried chicken, or fried fish?

- YES
- NO

» Yesterday, did you have any of the following beverages:

25 Tin milk or powdered milk?

- YES
- NO

26 Milo, tea with sugar, or coffee with sugar?

- YES
- NO

27 Fruit juice, fruit drinks, or sobolo?

- YES
- NO

28 Soft drinks or malts, such as, Coke, Fanta, Sprite, Alvaro, or Malta Guiness?

- YES
- NO

» Yesterday, did you get food from any place like...

29 KFC, Papaye, Pizza Inn, a mall food court, or other places that serve burgers or pizza?

- YES
- NO

DQQ Location

» DQQ Location - Healthy

Now, I would like to ask you where you were yesterday, when you consumed certain foods. You can answer with multiple options. Please State YES or NO, after each location.

- OK

Yesterday, where did you consume Kenkey, banku, tou zaafi, Hausa koko, akple, roasted maize, boiled maize, or tom brown?<br>*FOR ENUMERATOR: AT LEAST ONE MUST BE YES*		YES	NO
At Home	*	○	○
In School	*	○	○
Around School	*	○	○
On the go	*	○	○

Yesterday, where did you consume Beans, or bambara beans?<br>*FOR ENUMERATOR: AT LEAST ONE MUST BE YES*		YES	NO
At Home	*	○	○
In School	*	○	○
Around School	*	○	○
On the go	*	○	○

Yesterday, where did you consume Carrots, or sweet potatoes that are orange inside?<br>*FOR ENUMERATOR: AT LEAST ONE MUST BE YES*		YES	NO
At Home	*	○	○
In School	*	○	○
Around School	*	○	○
On the go	*	○	○

Yesterday, where did you consume Cocoyam leaves, amaranth leaves, ademe, ayoyo, potato leaves, cassava leaves, or bokoboko? *FOR ENUMERATOR: AT LEAST ONE MUST BE YES*		YES	NO
At Home	*	○	○
In School	*	○	○
Around School	*	○	○
On the go	*	○	○

Yesterday, where did you consume Baobab leaves, cowpea leaves, roselle leaves or bra, kenaf, African eggplant leaves, or West India nettle? *FOR ENUMERATOR: AT LEAST ONE MUST BE YES*		YES	NO
At Home	*	○	○
In School	*	○	○
Around School	*	○	○
On the go	*	○	○

Yesterday, where did you consume Tomatoes, okro, garden eggs, or cabbage? *FOR ENUMERATOR: AT LEAST ONE MUST BE YES*		YES	NO
At Home	*	◯	◯
In School	*	◯	◯
Around School	*	◯	◯
On the go	*	◯	◯

Yesterday, where did you consume Sweet green pepper, lettuce, cucumber, or mushrooms? *FOR ENUMERATOR: AT LEAST ONE MUST BE YES*		YES	NO
At Home	*	◯	◯
In School	*	◯	◯
Around School	*	◯	◯
On the go	*	◯	◯

Yesterday, where did you consume Mango, papaya, or African star apple? *FOR ENUMERATOR: AT LEAST ONE MUST BE YES*		YES	NO
At Home	*	◯	◯
In School	*	◯	◯
Around School	*	◯	◯
On the go	*	◯	◯

Yesterday, where did you consume Orange or tangerine?<br>*FOR ENUMERATOR: AT LEAST ONE MUST BE YES*		YES	NO
At Home	*	○	○
In School	*	○	○
Around School	*	○	○
On the go	*	○	○

Yesterday, where did you consume Banana, pineapple, avocado pear, watermelon, apple, or guava?<br>*FOR ENUMERATOR: AT LEAST ONE MUST BE YES*		YES	NO
At Home	*	○	○
In School	*	○	○
Around School	*	○	○
On the go	*	○	○

Yesterday, where did you consume Soursop, coconut, velvet tamarind, baobab, ebony fruit, or shea fruit?<br>*FOR ENUMERATOR: AT LEAST ONE MUST BE YES*		YES	NO
At Home	*	○	○
In School	*	○	○
Around School	*	○	○
On the go	*	○	○

Yesterday, where did you consume Groundnuts, kuli kuli, groundnut paste, groundnut soup, agushi stew, neri soup, or cashews? *FOR ENUMERATOR: AT LEAST ONE MUST BE YES*		YES	NO
At Home	*	○	○
In School	*	○	○
Around School	*	○	○
On the go	*	○	○

» DQQ Location Unhealthy

Yesterday, where did you consume Cakes, biscuits, rock bun, toogbee or bofrot? *FOR ENUMERATOR: AT LEAST ONE MUST BE YES*		YES	NO
At Home	*	○	○
In School	*	○	○
Around School	*	○	○
On the go	*	○	○

Yesterday, where did you consume Sausages or corned beef?<br>*FOR ENUMERATOR: AT LEAST ONE MUST BE YES*		YES	NO
At Home	*	○	○
In School	*	○	○
Around School	*	○	○
On the go	*	○	○

Yesterday, where did you consume Beef, goat, sheep, liver, or intestine?<br>*FOR ENUMERATOR: AT LEAST ONE MUST BE YES*		YES	NO
At Home	*	○	○
In School	*	○	○
Around School	*	○	○
On the go	*	○	○

Yesterday, where did you consume Pork , grasscutter , rabbit, or bush meat?<br>*FOR ENUMERATOR: AT LEAST ONE MUST BE YES*		YES	NO
At Home	*	○	○
In School	*	○	○
Around School	*	○	○
On the go	*	○	○

Yesterday, where did you consume Packaged yellow plantain chips or potato chips or Pringles?<br>*FOR ENUMERATOR: AT LEAST ONE MUST BE YES*		YES	NO
At Home	*	○	○
In School	*	○	○
Around School	*	○	○
On the go	*	○	○

Yesterday, where did you consume Indomie?<br>*FOR ENUMERATOR: AT LEAST ONE MUST BE YES*		YES	NO
At Home	*	○	○
In School	*	○	○
Around School	*	○	○
On the go	*	○	○

Yesterday, where did you consume French fries, fried yam, fried potato, atomo, spring rolls, fried chicken, or fried fish?<br>*FOR ENUMERATOR: AT LEAST ONE MUST BE YES*		YES	NO
At Home	*	○	○
In School	*	○	○
Around School	*	○	○
On the go	*	○	○

Yesterday, where did you consume Soft drinks or malts, such as, Coke, Fanta, Sprite, Alvaro, or Malta Guiness? *FOR ENUMERATOR: AT LEAST ONE MUST BE YES*		YES	NO
At Home	*	◯	◯
In School	*	◯	◯
Around School	*	◯	◯
On the go	*	◯	◯

Yesterday, where did you consume KFC, Papaye, Pizza Inn, a mall food court, or other places that serve burgers or pizza? *FOR ENUMERATOR: AT LEAST ONE MUST BE YES*		YES	NO
At Home	*	◯	◯
In School	*	◯	◯
Around School	*	◯	◯
On the go	*	◯	◯

Food Budget and in-kind consumption

In this section, we want to understand how much money you spend on certain foods. This is all money you have available, be it money you earned, pocket money or money you received from your parents to pay for food.

◯ OK

» In-Kind and eating practices

Did you carry your own food from home yesterday?

- Yes, my breakfast
- Yes, my lunch
- Yes, a snack
- No

Did you have breakfast yesterday?

- Yes, in school
- Yes, around school or on the go
- Yes, at home
- No, I did not have breakfast

Did you have lunch yesterday?

- Yes, in school
- Yes, around school or on the go
- Yes, at home
- No, I did not have lunch

» Budget

How much money did you spend on food yesterday?

In Cedis, this is the money you spent yourself, but it can be money that someone gave you.

How many Cedis did you spend yesterday on... BREAKFAST?

Enter 0, if breakfast was provided.

How many Cedis did you spend yesterday on... LUNCH?

How many Cedis did you spend yesterday on... SNACKS?

KAP

KAP - Introduction:

I will now ask you a few questions about what you think about eating and when you share meals together. This section is about what YOU know about these things, so please answer truthfully. This is not a test and all your answers will remain anonymous and won't be shared with others.

- OK

» PRACTICES

In the following questions, you will be asked about your typical school week, going from Monday to Friday. Think about the last 5 school days, even if they were separated by the weekend and please tell me on how many days (out of five) the following applies:

» » Personal Eating Habits

In a normal school week (5 days) how often do you have breakfast before leaving for school?

- 1
- 2
- 3
- 4
- 5
- 0

In a normal school week (5 days) how often do you bring your lunch to school?

- 1
- 2
- 3
- 4
- 5
- 0

In a normal school week (5 days) on how many days do you skip a meal?

This can be one or more meals per day, relevant is the number of days.

- 1
- 2
- 3
- 4
- 5
- 0

» » Readiness to Change & Self-Efficacy

For the next section, I will ask you to respond with either YES or NO to the statement.

I try to eat breakfast everyday

- YES
- NO
- DON'T KNOW

I try to eat two pieces of fruit everyday

- YES
- NO
- DON'T KNOW

I try to drink less soft drinks

- YES
- NO
- DON'T KNOW

» » Peer Group dietary practices

Finally, I would like to ask about how often you eat with friends and family in a typical week - regardless of school days or not. I will therefore ask you to think about the last 7 days and answer how often the following scenarios occurred:

In the last 7 days, how often did you and your friends - eat breakfast together away from home

- 1
- 2
- 3
- 4
- 5
- 6
- 7
- 0

In the last 7 days, how often did you and your friends - eat a mid-morning snack together

This means eating at the same time, it does not need to be sharing. It can be a toffee or bogfrot or a piece of fruit.

- 1
- 2
- 3
- 4
- 5
- 6
- 7
- 0

In the last 7 days, how often did you and your friends - eat lunch together away from home / at or around school

- 1
- 2
- 3
- 4
- 5
- 6
- 7
- 0

In the last 7 days, how often did you and your friends - eat dinner together away from home

- 1
- 2
- 3
- 4
- 5
- 6
- 7
- 0

» » Family dietary practices

In the last 7 days, how often did you and your FAMILY - eat out at a restaurant/ order take away?

- 1
- 2
- 3
- 4
- 5
- 6
- 7
- 0

In the last 7 days, how often did you and your FAMILY - eat a home cooked evening meal together at home?

- 1
- 2
- 3
- 4
- 5
- 6
- 7
- 0

In the last 7 days, how often did you and your FAMILY - eat breakfast together at home?

- 1
- 2
- 3
- 4
- 5
- 6
- 7
- 0

» KNOWLEDGE

» » Eating Habits

» » » Breakfast

Some children do not have breakfast before going to school and are hungry in class. What is the consequence for children of not having breakfast and being hungry at school?

- Children have short attention/have low concentration/cannot study well/ do not do as well at school as they should
- Other
- Don't know

Specify, if other:

» » » Sugar-rich foods

Why should parents discourage sticky and sugar-rich foods, such as sweets and candies? (Why is it so bad to eat too many sweets and candies?)

- Because they can cause tooth decay
- Because they are not nutritious
- Because they interfere with appetite
- Other
- Don't know

Specify, if other:

» » » SSB

What are healthy alternatives to Sugar-Sweetened Beverages?

- ☐ Water or Fruit Juice
- ☐ Other
- ☐ Don't know

Specify, if other:

» » NCDs

» » » Overweight

What are the health problems that can occur when a person is overweight or obese?

- ☐ Increased risk of chronic conditions (such as heart/cardiovascular disease, high blood pressure and diabetes, stroke, certain types of cancer, respiratory difficulties, chronic musculoskeletal problems, skin problems and infertility)
- ☐ Reduced quality of life
- ☐ Premature death
- ☐ Other
- ☐ Don't know

Specify, if other:

» » » Overweight Prevention

How can people prevent overweight and obesity?

- [ ] Reduce energy intake (less high-energy foods and drinks)/reduce the intake of fatty and sugary foods
- [ ] Eat vegetables and fruits more often
- [ ] Eat legumes/whole-grain products more often
- [ ] Increase physical activity level/engage in regular physical activity
- [ ] Other
- [ ] Don't know

Specify, if other:

» » Food Groups

» » » Food Group Plate

Which food groups should take the most space on your plate?

- [ ] Vegetables (okro, garden eggs, tomatoes, aleefu, ayoyo, kontomire, cassava leaves, sweet potato leaves, onions, green beans, cabbage, carrots, lettuce, bitter leaves, green pea, garden eggs (kwawunsunsuaa))
- [ ] Fresh Fruit (Orange, pawpaw, mango, watermelon, pineapple, apple, grapefruit, Alaasa, lemon, banana,)
- [ ] Starches (Cereals (rice, maize, millet, sorghum, wheat), Plantain, Roots (yam, cassava, cocoyam, sweet potatoes))
- [ ] Other
- [ ] Don't know

Specify, if other:

» » » Servings of fruit and veg

How many servings of fruit AND vegetables should children consume per day?

This is both fruit AND vegetables

- 1 to 2
- 3 to 4
- 5 to 6
- 7 to 8
- Other
- Don't know

Specify, if other:

» » » Smallest part of diet

Which foods should make up the smallest part of your diet?

For Enumerator: As sugar can be taken with drinks, this refers to total diet, where the previous one was plate :)

- Sugar
- Fats
- Other
- Don't know

Specify, if other:

» ATTITUDE

All the following items consist of the options "agree very much, agree, neither agree nor disagree, disagree or disagree very much". I would like you to listen to the question first and then state, which of these options applies.

FOR ENUMERATOR - TRY TO READ OUT ONLY THE QUESTION OR ITEM AND GET RESPONSE. AVOID READING OUT ALL THE OPTIONS EVERYTIME.

» » Perceived susceptibility and severity

I am worried about becoming Overweight or Obese

- Agree very much
- Agree
- Neither Agree nor Disagree
- Disagree
- Disagree very much
- DO NOT KNOW

I am worried about getting Diabetes

- Agree very much
- Agree
- Neither Agree nor Disagree
- Disagree
- Disagree very much
- DO NOT KNOW

» » Perceived Benefits

Eating fruits can help you fight infections

- Agree very much
- Agree
- Neither Agree nor Disagree
- Disagree
- Disagree very much
- DO NOT KNOW

Eating vegetables can help you lose weight

- Agree very much
- Agree
- Neither Agree nor Disagree
- Disagree
- Disagree very much
- DO NOT KNOW

Regular breakfast helps improve being alert

- Agree very much
- Agree
- Neither Agree nor Disagree
- Disagree
- Disagree very much
- DO NOT KNOW

» » Perceived Barriers

I am not sure what I should eat for a healthy diet

- Agree very much
- Agree
- Neither Agree nor Disagree
- Disagree
- Disagree very much
- DO NOT KNOW

I typically do not have enough money for eating fresh fruits

- Agree very much
- Agree
- Neither Agree nor Disagree
- Disagree
- Disagree very much
- DO NOT KNOW

There are no healthy food options offered in and around school

- Agree very much
- Agree
- Neither Agree nor Disagree
- Disagree
- Disagree very much
- DO NOT KNOW

DONE - THANK YOU VERY MUCH FOR YOUR PARTICIPATION.

As a thank you for your time, please accept this token of appreciation and we wish you all the best for your future.
