# Supplementary material for: Individual‐Level Drivers of Food Choices and Diet Quality Among Adolescents in Urban West Africa: Evidence From Accra, Ghana
Source: Matern Child Nutr. 2024 Dec 9;21(2):e13775. doi: 10.1111/mcn.13775 (PMC11956040; doi:10.1111/mcn.13775)
Supplement: Supplementary file 2 — Supporting information. [file MCN-21-e13775-s002.docx]

Supplementary Material I

**Table SI**

**Categorization of independent variables used according to different food environment frameworks: 1) Fanzo et al. (2020) adapted from HLPE 2) Turner et al. 2018 3) Osei-Kwasi et al. (2020)**

Although not formatively used for the design of this study, these dimensions align with the factors outlined for the African urban food environment regarding individual (demographic, cognitions, practices) and social-environment (family, friends, societal) (Osei-Kwasi et al., 2020). They are also aligned with the personal domain aspects of the 2018 food environment framework (Turner et al., 2018).

| 1 - FSD | Economic | Cognitive | Aspirational | Situational | Consumer Behaviour |
| --- | --- | --- | --- | --- | --- |
| 2 – Turner | Affordability | Desirability | | Convenience | |
| 3 – AU FE | Demographic | Cognitions | Cognitions | Social-Environment | Practices |
| Predictors | *Food Budget* | *Knowledge about eating practices* | *Perceived Susceptibility* | *Family eating habits* | *Readiness to Change* |
|  | *Household Assets* | *Knowledge about NCDs* | *Perceived Benefits* | *Peer eating habits* | *Eating Patterns* |
|  |  | *Knowledge about Food Groups* | *Perceived Risks* |  |  |
